# Effect of Al_2_Ca Addition and Heat Treatment on the Microstructure Modification and Tensile Properties of Hypo-Eutectic Al–Mg–Si Alloys

**DOI:** 10.3390/ma14164588

**Published:** 2021-08-16

**Authors:** Abdul Wahid Shah, Seong-Ho Ha, Bong-Hwan Kim, Young-Ok Yoon, Hyun-Kyu Lim, Shae K. Kim

**Affiliations:** 1 Industrial Technology Department, University of Science and Technology, Daejeon 34113, Korea; shae@kitech.re.kr; 2 Advanced Process and Materials R&D Group, Korea Institute of Industrial Technology, Incheon 21999, Korea; shha@kitech.re.kr (S.-H.H.); bongk75@kitech.re.kr (B.-H.K.); veryoon@kitech.re.kr (Y.-O.Y.); hklim@kitech.re.kr (H.-K.L.)

**Keywords:** Al–Mg–Si alloys, Al_2_C addition, microstructure, heat treatment, tensile strength

## Abstract

The current study investigated the microstructure modification in Al–6Mg–5Si–0.15Ti alloy (in mass %) through the minor addition of Ca using Mg + Al_2_Ca master alloy and heat treatment to see their impact on mechanical properties. The microstructure of unmodified alloy (without Ca) consisted of primary Al, primary Mg_2_Si, binary eutectic Al–Mg_2_Si, ternary eutectic Al–Mg_2_Si–Si, and iron-bearing phases. The addition of 0.05 wt% Ca resulted in significant microstructure refinement. In addition to refinement, lamellar to fibrous-type modification of binary eutectic Al–Mg_2_Si phases was also achieved in Ca-added (modified) alloy. This modification was related to increasing Ca-based intermetallics/compounds in the modified alloy that acted as nucleation sites for binary eutectic Al–Mg_2_Si phases. The dendritic refinement with Ca addition was related to the fact that it improves the efficacy of Ti-based particles (TiAl_3_ and TiB_2_) in the melt to act as nucleation sites. In contrast, the occupation of oxide bifilms by Ca-based phases is expected to force the iron-bearing phases (as iron-bearing phases nucleate at oxide films) to solidify at lower temperatures, thus reducing their size. The as-cast microstructure of these alloys was further modified by subjecting them to solution treatment at 540 °C for 6 h, which broke the eutectic structure and redistributed Mg_2_Si and Si phases in Al-matrix. Subsequent aging treatment caused a dramatic increase in the tensile strength of these alloys, and tensile strength of 291 MPa (with El% of 0.45%) and 327 MPa (with El% of 0.76%) was achieved for the unmodified alloy and modified alloy, respectively. Higher tensile strength and elongation of the modified alloy than unmodified alloy was attributed to refined dendritic structure and modified second phases.

## 1. Introduction

Metal matrix composites (MMCs) were recently extensively used as structural materials in various industries, e.g., automotive, aerospace, defense, marine, oil, and electronic [1]. Aluminum (Al) and its alloys are widely used as the matrix material for manufacturing these MMCs because of the unique characteristics associated with them, such as low density, good corrosion resistance, and high thermal/electrical conductivity [1,2]. Due to their better performance and longer life, aluminum matrix composites (AMCs) are considered promising materials to replace their conventional casting alloy counterparts in many applications [1,2,3]. These AMCs are produced via different processing methods such as diffusion bonding, powder metallurgy, and casting process [1,2]. Among them, the casting processing route is considered the easiest and efficient way to produce complex shapes. In the casting process, the reinforced particles can be introduced in the Al-matrix through in situ or ex situ methods. Most of the commercial AMCs are fabricated through the ex situ method. However, one of the hurdles in achieving the real potential of ex situ AMCs is the processing of ceramic-particle-containing melt as the casting of these ex situ composites is difficult as these solid particles in the melt decreased fluidity of the molten metal [1,2,3]. As a result, their processing method is more complicated and expensive compared to conventional casting processes. Therefore, for the last few decades, research has focused on developing in situ composites to make the processing of these composites easy while giving equal or better final properties than that achieved in ex situ composites [1,2,3,4,5,6,7,8,9,10,11]. In the in situ composites, precipitation of reinforced phase occurs during the solidification process, and therefore, the shape and size of these particles can be tailored to some extent by controlling the cooling rate or introducing additives in the melt [1,2,3,4,5].

When it comes to in situ aluminum composites, the Al–Mg–Si system is considered a potential candidate. The Al–Mg_2_Si based in situ composites are considered a promising material for wear resistance and high-temperature applications, where hyper-eutectic Al–Si alloys (such as A390) and the Al–SiC composites are currently being used [1,2,3,4,5,6,7,8,9,10]. The Mg_2_Si intermetallic compound is characterized by a high melting point, high thermal stability accompanied by low thermal expansion, low density of 1.95 g/cm^3^, and high mechanical properties. Further, the interface bonding between the Mg_2_Si intermetallic compound and the aluminum matrix is strong and is considered the most important criterion for the overall strength of a given AMC. In the past, the development of Mg_2_Si reinforced Al-matrix in situ composites was extensively investigated. Most of these studies were performed on the hyper-eutectic Al–Mg_2_Si pseudo-binary compositions. However, a problem with hyper-eutectic Al–Mg_2_Si in situ composites is the coarse size of the primary Mg_2_Si phases. These coarser Mg_2_Si phases have a damaging effect on the mechanical properties of these in situ composites [4,5,6]. Therefore, many studies were conducted to enhance the mechanical properties of Al–Mg_2_Si composites by refining the primary Mg_2_Si by introducing modifier elements [6,7,8,9,10]. Ghandvar [4] studied the effect of simultaneous addition of Gd and Sb on the microstructure and corresponding mechanical properties of Al–15%Mg_2_Si composite. This resulted in a significant decrease in the size of primary Mg_2_Si phases compared to the base composition. Similarly, Qin [7] and Khorshidi [8] reported morphological changes in primary Mg_2_Si phases through the addition of yttrium (Y) and lithium (Li), respectively, which in turn led to some enhancement in tensile strength and elongation. In the same way, Li [9] and Nasiri [10] found the refinement of primary Mg_2_Si phases through the addition of phosphorous (P) in Al–Mg–Si hyper-eutectic alloys, and as a result, improvement in tensile strength and elongation was achieved. However, although morphological changes and some size reduction in primary Mg_2_Si phases through additive addition, no significant increase in the tensile strength of the modified hyper-eutectic Al–Mg_2_Si in situ composites has been achieved thus far.

The alternative way to avoid the formation of coarse primary Mg_2_Si phases could be through developing these Al–Mg_2_Si in situ composites based on hypo-eutectic composition (eutectic point at 13.9 wt% Mg_2_Si). A pseudo-binary phase diagram of Al–Mg_2_Si can be found elsewhere [5]. The hypo-eutectic Al–Mg_2_Si alloys mostly consist of eutectic Al–Mg_2_Si binary phases with no or small amount of polygonal-shaped primary Mg_2_Si phases [5,11,12,13]. Ji [11] developed a hypo-eutectic Al–Mg_2_Si alloy (with approximately 4 wt% Mg_2_Si) based novel die casting alloy with high strength accompanied by high ductility. Following that, few studies were conducted regarding the evolution of eutectic microstructure in this newly developed alloy [14,15,16,17,18]. Zhu [5] recently studied the effect of varying amounts of extra Si and Mg on eutectic phases in Al–Mg_2_Si alloy processed by high pressure die casting. In this study, different eutectic Mg_2_Si morphologies were reported, such as rod, flake, lamellar. However, there is a lack of complete understanding regarding the nucleation and growth of these various eutectic morphologies and mechanisms related to the refinement/modification through additive addition. Therefore, the current study investigated the microstructural evaluation and its modification in Al–6Mg–5Si–0.15Ti alloy (in mass %) through the minor addition of Ca (using Mg + Al_2_Ca master alloy) and heat treatment and corresponding changes in mechanical properties.

## 2. Materials and Methods

The chemical composition of the experimental alloys is shown in Table 1. High purity (99.99%) aluminum (Al) ingots were used in this study. The addition of magnesium (Mg), silicon (Si), and titanium (Ti) in the melt was conducted in the form of pure Mg, Al–25 mass%Si master alloy (analyzed composition is provided in Table 2), and Al–5 mass%Ti–1 mass%B master alloy, respectively. Additionally, Mg + Al_2_Ca master alloy was employed for the modified alloy to introduce Mg and Ca in the melt. In the melting and alloying process, first pure Al was melted in the induction furnace at ambient atmosphere. Once the Al melted down and the temperature reached ~780 °C, alloying was conducted for the respective alloys. After that, the melt was held at ~750 °C for a few minutes to achieve the uniform distribution of alloying elements in the melt. Afterward, melt treatment, using the Ar-based gas bubbling filtration process, was employed for 15 min to remove hydrogen gas and oxide inclusions. The temperature during the degassing process was maintained at ~700 °C. Once the degassing process was completed, melts were held at ~690 °C for 5 min before pouring into the steel mold, preheated to 200 °C. The pouring for all examined alloys was executed at 690 °C.

The surface of each sample was ground, micro-polished, and etched in Keller’s reagent for microstructure observation. To observe the 3D morphology and size of the primary and eutectic Mg_2_Si phases, deep etching was performed in a solution of 5% HCl and 95% ethanol. Each specimen was etched for 300 min followed by ultrasound cleaning in ethanol solution to remove the left etchant completely from the surface. Optical microscopy (OM) (Nikon, Tokyo, Japan) and field emission-scanning electron microscopy (FE-SEM, FEI model Quanta 200 F, FEI, Hillsboro, OR, USA) coupled with energy dispersive spectroscopy (EDS, EDAX, Pleasanton, CA, USA) were employed for the microstructure observation. The FESEM analysis was performed under the accelerating voltage of 20 KV with a working distance of 10.0 mm. The heat treatment process began with subjecting the examined alloys to the solution treatment at 540 °C for 6 h, followed by water quenching. Subsequently, these solution heat-treated samples underwent aging treatment in the aging furnace at 190 °C for 10 h. A Brinell hardness machine(Buehler, Uzwil, Switzerland) (using B scale) was used to measure the hardness of aged samples with varying aging times. Five measurements were taken for each specimen at each condition, and an average of these five values is reported here. Additionally, microstructural characterization of examined alloys in as-quenched and peak hardness tempers was also conducted to investigate the microstructure modification upon heat treatment. The samples for the tensile test were prepared according to ASTM standard B557, and a universal tensile testing machine (Model number DTU-900MHN, Daekyung Tech, Gumisi, South korea) was employed to perform tensile testing. The gauge length of the extensometer was 30 mm, and a strain rate of 1.5 mm/min was applied during all tensile tests. The precipitation hardening behavior of the examined alloys was investigated through differential scanning calorimetry (DSC, TA Q1000 instrument, TA instruments, Milford, MA, USA). In thermal analysis, examined alloys in as-quenched (SHT), peak hardness, and overaged states were studied. The samples were placed in pure aluminum pans for heating in the furnace, and each sample was heated between 50 °C and 580 °C under an argon atmosphere at a rate of 10 °C per minute. Optical emission spectroscopy (OES, Bruker model Q2 ION, Bruker, Billerica, MA, USA) was employed to determine the composition of the Al–25 mass%Si master alloy.

## 3. Results and Discussion

**Figure 1:**
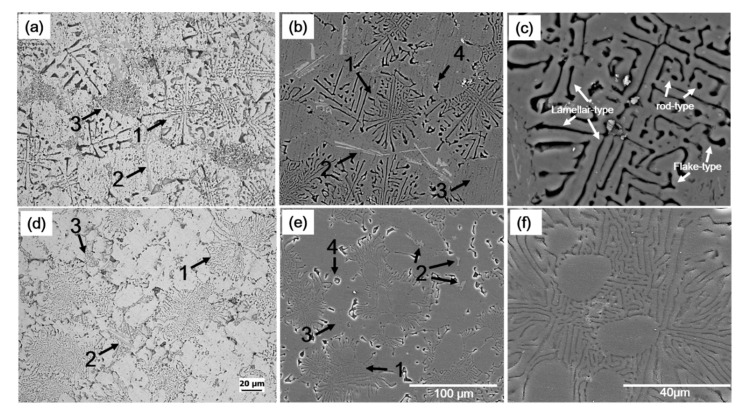
As-cast microstructures of unmodified (A1) and modified (E1) alloys; (**a**,**d**) are optical micrographs of A1 and E1 alloy, respectively. Whereas (**b**,**c**) are SEM micrographs of A1 alloy at different magnifications, and (**e**,**f**) are showing SEM micrographs of E1 alloy at varying magnifications. Here 1, 2, 3, and 4 indicate the binary eutectic Al–Mg2Si phases, iron-bearing phases, ternary eutectic Al–Mg2Si–Si phases, and primary Mg2Si phases, respectively.

**Figure 2:**
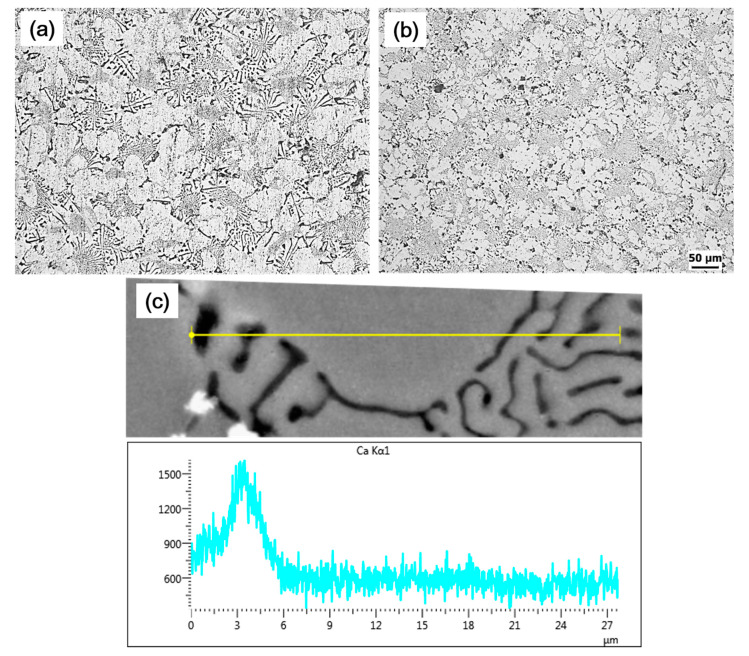
Optical microstructure of (**a**) A1 alloy and (**b**) E1 alloy at relatively 200× magnification representing the relative size of SDAS in examined alloys. (**c**) It shows the SEM–EDS (line scan) analysis result presenting the Ca composition variation across the dendrite and interdendritic region in E1 alloy.

The as-cast microstructures of the experimental alloys are shown in Figure 1. The microstructures of unmodified alloy (hereinafter called A1 alloy) consisted of α-Al phase, primary Mg_2_Si, eutectic Al–Mg_2_Si binary, eutectic Al–Mg_2_Si–Si ternary, and iron-bearing phases (Figure 1a,b). The primary Mg_2_Si phases (#4 in Figure 1) were mostly found in the polygonal shapes, and their size was much smaller than that reported in hyper-eutectic alloys [4,5,6,7,8,9,10]. The lamellar eutectic structure (#1 in Figure 1) was the dominant morphology of eutectic binary Al–Mg_2_Si phases in the A1 alloy along with rod-type and flake-like morphology (Figure 1c). According to theoretical calculations [12,13,14,15], these binary eutectic phases formed due to a univariant pseudo-binary eutectic reaction. A more refined structure of eutectic Al–Mg_2_Si–Si ternary phases (#3 in Figure 1) was observed than that of the lamellar eutectic structure of binary eutectic phases, which is believed to be formed because of an invariant ternary reaction at the end of solidification [12,13]. In addition to these phases, the iron-bearing phases (#2 in Figure 1) were also present, and most of these phases with mostly plate or needle morphology were present within or along with the eutectic Al–Mg_2_Si–Si ternary phases. The modified alloy (hereinafter called E1 alloy) had the same phases as in the A1 alloy (Figure 1d,e); however, the addition of Ca had significantly refined these phases in E1 alloy (Figure 1d,e) compared to A1 alloy (Figure 1a–c). Additionally, it was also observed that Ca addition led to a significant decrease in the size of secondary dendritic arm spacing (SDAS) in E1 alloy (Figure 2b) than that in A1 alloy (Figure 2a). SDAS of ~50 µm in A1 alloy decreased down to ~20 µm in E1 alloy.

**Figure 3:**
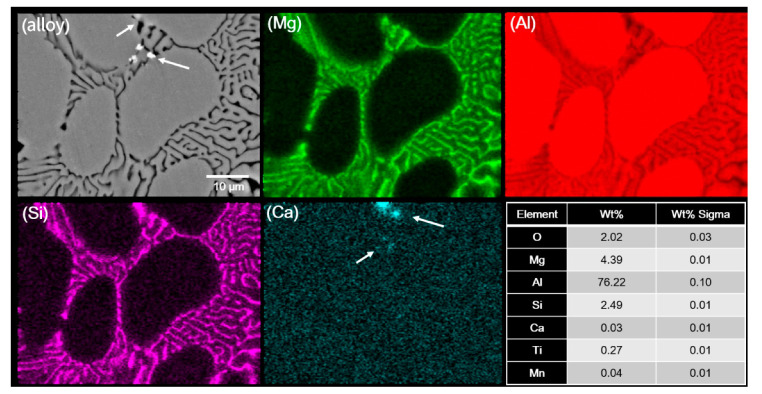
SEM–EDS mapping analysis of E1 alloy revealing the composition distribution of various elements. (alloy) SEM micrograph of E1 alloy, while the table shows the composition of various elements detected in mapping analysis. Here, white arrows are pointing at Ca-rich phases.

Ca-induced SDAS refinement of the primary α-Al phase was also reported in previous studies [19,20,21,22,23]. Zhang [22] reported a decrease in the dendritic size of A356 alloy upon the addition of 0.06 wt% Ca. It was stated that refinement was related to increased undercooling by the enrichment of Ca on the solid/liquid interface, and consequently, it restricted the growth of the primary α-Al phase. However, in another study [23], it was reported that Ca addition in inoculated Al–Si–Mg ternary alloys increases the nucleation of primary α-Al phases by promoting heterogeneous nucleation sites Al_3_Ti or Al_2_Cu, which in turn resulted in dendritic refinement. Similar to Jiao [23], Ravi [24] also suggested that the addition of elements with high oxidation tendency tends to increase the efficacy of Ti-based particles (TiAl_3_ and TiB_2_) by scavenging oxygen present on their surface and thus improves wettability as well as by reducing the agglomeration tendency of these particles. In this study, the elemental distribution analysis was performed using SEM–EDS analysis, as shown in (Figure 2c) and (Figure 3). SEM–EDS (line scan) analysis (Figure 2c) resulted in a relatively higher Ca content within the interdendritic region, which decreased significantly in the primary Al dendrite. However, an almost similar Ca was found within the interdendritic region located on the other side of the primary Al dendrite. Likewise, there was no significant variation in Ca content in EDS-mapping analysis, and only a few spots were observed where the Ca amount was higher. From these results, it seems that there was no significant segregation of Ca on the solid/liquid interface, and therefore, this cannot be the reason for grain refinement. However, in addition to Ca, Ti and Mn were also found at these Ca-rich spots, as shown in the table in Figure 3. Therefore, these white phases are believed to be Ti-based alloys with a significant amount of Ca. Moreover, Ca has a very high affinity for oxygen; therefore, the mechanism proposed by Ravi [24] and Jiao [23], which was related to improving the potency of nuclear particles in the melt, can be related to the dendritic refinement in the current study. That is, free Ca in the melt reacts to oxides present on Ti-based particles and scavenge oxygen from these particles. As a result, wettability was improved, as suggested by Ravi [24]. Therefore, it can be expected that dendritic refinement in Ca added alloy is related to the fact that it improves the efficacy of Ti particles in the melt.

**Figure 4:**
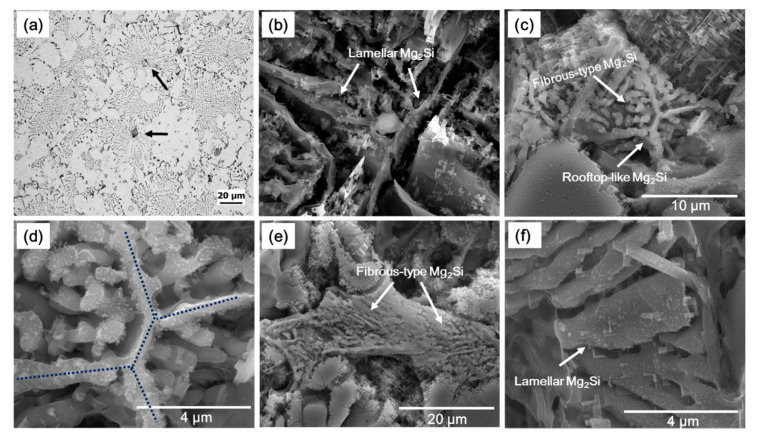
(**a**) optical micrograph of E1 alloy which Black arrows are pointing at the primary Mg2Si phases located in the middle of binary eutectic Al–Mg2Si phases. The rest are SEM microstructures, acquired using the SE mode, of deep-etched investigated alloys; (**b**) the coarse curved lamellar binary eutectic structure found in A1 alloy. (**c**) Low and (**d**) high magnification showing the rooftop-like Mg2Si phase in E1 alloy. (**e**,**f**) Fibrous-type modified and refined lamellar binary eutectic Al–Mg2Si phases in E1 alloy, respectively.

**Figure 5:**
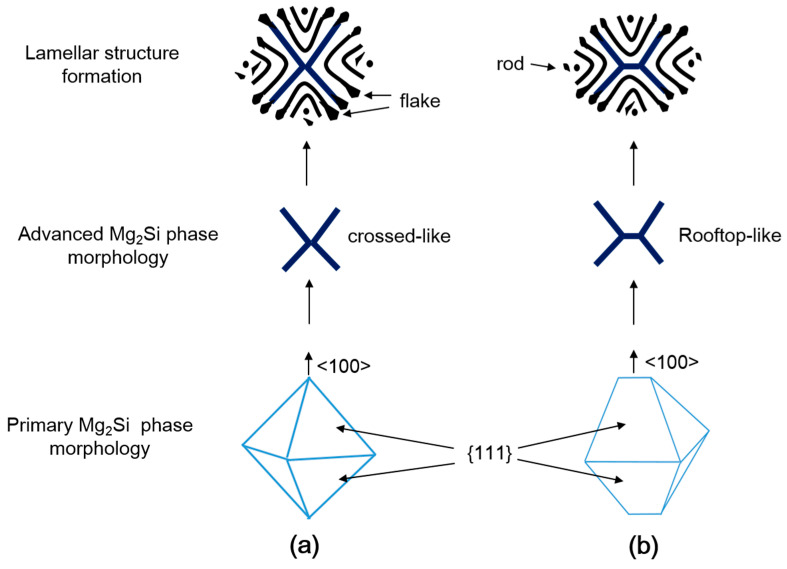
Schematic drawing to explain the mechanisms of evolution of eutectic phases from polygonal primary Mg2Si phases. (**a**) The formation of crossed-like advanced Mg2Si phase and subsequent lamellar structure formation upon it and (**b**) the evolution of lamellar structure from rooftop-like morphology of advanced Mg2Si phase (octahedral shapes are redrawn from ref. [15]).

According to previous studies [14,15,21], the primary Mg_2_Si phases are believed to act as nucleation sites to form eutectic Al–Mg_2_Si binary phases. This was also observed in the current study, where many primary Mg_2_Si phases were found in the center of the eutectic Al–Mg_2_Si binary phases, such as shown in Figure 4a. Li [15] reported three morphologies of eutectic Al–Mg_2_Si binary phases in hypo-eutectic alloys, with composition near the eutectic point, and reported them to originate from the octahedral shape primary Mg_2_Si phases. These morphologies were (1) rod-like, (2) crossed-like, and (3) rooftop-like. The octahedral shapes of primary Mg_2_Si phases suggested by Li [15] are redrawn in this study, as shown in Figure 5. It was stated that a rod-like morphology is obtained if the eutectic Mg_2_Si started forming from the vertex of the corner of an octahedral primary crystal. However, the nucleation of the eutectic Mg_2_Si phase at a vertex and the four edges of the octahedral may yield a crossed-type (Figure 5a), or a rooftop-like morphology is evolved if the octahedral is in shape shown in (Figure 5b). Vertices and edges of these octahedra are less stable than the faces, and as a result, heat is released more rapidly from the former positions. Therefore, the advanced Mg_2_Si, with the aforementioned morphologies, evolve from vertices <100> and edges <110> instead from faces {111} [21]. However, many recent studies related to hypo-eutectic Al–Mg–Si alloys reported the formation of lamellar eutectic structure as well as other morphologies such as Chinese script-like or flake-like morphology [11,17,18,21]. Trudonoshyn [21] also reported the triangular-spiral morphology. The SEM microstructures of currently examined alloys with deeply etched conditions are shown in Figure 4. Figure 4b shows the morphology of binary eutectic Mg_2_Si phases in the A1 alloy. The curved lamellar eutectic morphology with coarse Mg_2_Si phase lamellae is clear. This layer structure of binary eutectic Mg_2_Si phases shows a cross-like or rooftop-like structure in the middle from where the lamellar structure seemed to be evolved. Furthermore, the Mg_2_Si phase with rooftop-like morphology was also observed in the modified alloy (Figure 4c,d). From these results, it can be concluded that the formation of lamellar structure, which accounts for a majority of eutectic colonies, originates from the rooftop or cross-type, such as the advanced Mg_2_Si phase that evolved from the primary Mg_2_Si phases. This mechanism of lamellar (and subsequent rod-like and flake-like) eutectic structure formation from the polygonal primary Mg_2_Si phases is schematically drawn in Figure 5. The further growth of univariant eutectic phases from crossed-like (Figure 5a) or rooftop-like (Figure 5b) advanced Mg_2_Si phase occurs through the formation and growth of lamellar structure perpendicular to advancing front. However, as the lamellar structure proceeds, due to some changes in the interface energy, the morphology of eutectic phases changed from lamellar to rod-like or flake-like. Ca addition refined the eutectic structure, but it also led to the morphological modification of pseudo-binary eutectic phases from lamellar structure (Figure 4b) to fibrous (Figure 4e). However, not all the eutectic phases were modified to fibrous, and there was still a lamellar eutectic structure (Figure 4d) in the modified alloy but with significant refinement. Similar modification of Al–Si eutectic structure upon the addition of various amounts of Ca was also reported in previous studies [22,23]. Zhang [22] reported an addition of 0.06 wt% Ca in A356 alloy to achieve full modification and corresponding enhancement in mechanical properties. However, in the currently investigated alloy, a higher amount of Ca is needed to achieve full modification.

**Figure 6:**
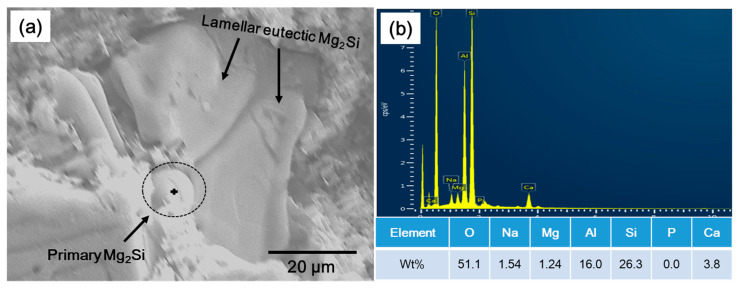
(**a**) SEM microstructure of deep-etched A1 alloy showing a coarse binary eutectic Al–Mg2Si phase and a primary Mg2Si phase in the middle of it. (**b**) It showed the EDS-analysis result of a point, indicated with a plus sign in (**a**), located on this central primary Mg2Si phase, which has shown the presence of Ca element in it.

From the literature, two mechanisms are responsible for modification/refinement of eutectic structure on the addition of a modifier element [16,18,19,22,23,24,25,26]; (1) The modifier element, either in the form of oxides or thermally stable intermetallic compounds, act as nucleation sites for eutectic phases or it should deactivate the potency of nucleating agents present in the melt (which are usually present in small amount as an impurity), and (2) is through restricting the growth of these phases by changing the interface energy of solid/liquid interface. In the case of Al–Mg–Si ternary alloys, different nucleation sites are reported for the primary Mg_2_Si phases, upon which eutectic phases are formed. One study reported that these phases are heterogeneously nucleate at oxides present in the melt [21]. However, Pabel [27] reported Ca-containing intermetallic compounds (CaMg_2_, Al_2_Ca, and Al_4_Ca) that nucleate on oxide inclusions and later act as nucleation sites for Mg_2_Si phases. This study [27] was related to the modifying and refinement of eutectic phases in as-cast Al–Mg–Si alloys with phosphorus (P). It was reported that P reacts with Ca and, in this way, decrease the Ca-containing intermetallic compounds. Zhang [22], in his work, reported the formation of these intermetallic compounds when both Ca and P are present in the melt; Ca_3_(PO_4_)_2_ with a melting point of 1391 °C, and Ca_3_P_2_ with a melting point of 1600 °C. It is further stated that Ca_3_(PO_4_)_2_ has a high density of 31.8 g/cm^3^, and because of this, it settles down at the bottom. Alternatively, Ca_3_P_2_, with a density of just 2.51 g/cm^3^, appears on the melt surface, which is removed during the skimming process. Thus P addition leads to the removal of Ca-containing intermetallic compounds, such as CaMg_2_, Al_2_Ca, and Al_4_Ca, potent nucleation sites for Mg_2_Si phases. According to Pabel [27], this scenario forced Mg_2_Si primary phases to form on the primary α-Al instead, considered a poor nucleating agent. As follows, the formation temperature of eutectic phases decreases and, as a result, modifies the eutectic structure. A similar explanation was provided by Campbell [20] regarding the refinement of Si eutectic phases in Al–Si alloys by the addition of Sr. In Al–Si alloy, AlP particles are considered nucleating agents for Si eutectic phases. It was reported that Sr decreased the potency of AlP and oxide films to act as nucleating agents for Si eutectic phases and therefore decrease the eutectic formation temperature leading to refinement and modification of Si plates. In a study related to hyper-eutectic Al–Si alloys [26], Al-Halal reported a significant modification and refinement of Si eutectic phases in the Al–15Si alloy upon addition of 0.5%Ca. This refinement was associated with the formation of Al_2_CaSi_2_ phases, which is reported to form on the oxide films, and consequently, become the nucleation sites for Si in Al–Si alloys. In the current study, EDS analysis of a primary Mg_2_Si phase located in the middle of a eutectic colony revealed the Ca element in A1 alloy (with 0.0012 wt% Ca), shown in Figure 6. Therefore, these observations, along with the abovementioned previous studies [26,27], lead us to believe that Ca-containing compounds are the potential nucleation sites for the primary Mg_2_Si phases and, as a result, influence the formation of eutectic phases. Therefore, the refinement of eutectic phases in E1 alloy in the current study can be related to the increasing number of these Ca-containing intermetallic compounds in the melt that acted as nucleation sites for the primary Mg_2_Si phases. A large number of these primary phases mean increasing nucleation sites for the binary eutectic phases, which led to a refinement of these phases in the modified alloy. On the other hand, for lamellar to fibrous-type eutectic modification, Trudonoshyn [17] reported that modification (lamellar to fibrous-type) of binary eutectic Al–Mg_2_Si structure is related to nucleation of these phases directly on inoculated particles, instead of primary Mg_2_Si phases. However, in the current study, the fibrous-type eutectic phases were observed around the rooftop-like advanced Mg_2_Si phases (Figure 4c,d). This shows that the fibrous-type eutectic structure forms after the formation of rooftop-like or crossed-like advanced Mg_2_Si phases. Therefore, as reported by Trudonoshyn [17], lamellar to fibrous-type modification after these advanced Mg_2_Si phases can be related to the nucleation of these phases directly on the Ca-based particles resulting in the fibrous structure.

**Figure 7:**
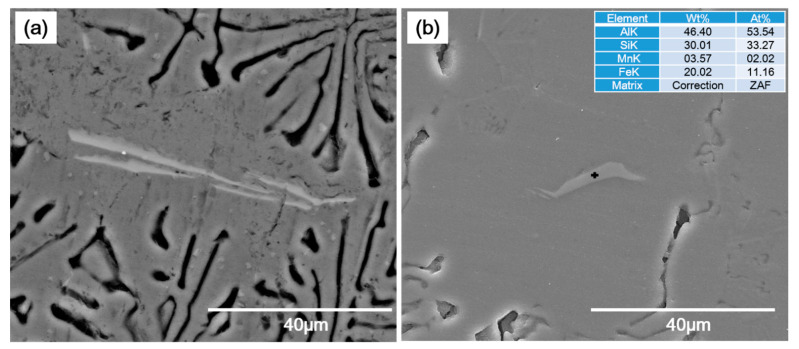
SEM microstructures of as-cast A1 alloy (**a**) and E1 alloy (**b**), showing the relative size of iron-bearing phases in these investigated alloys. A table on the right upper corner shows the EDS-analysis result of the point indicated with a plus sign in (**b**).

A significant decrease in the size of iron-bearing phases was also observed upon the addition of Ca in E1 alloy (Figure 7b) if their size is compared with ones present in A1 alloy (Figure 7a). The iron-bearing plate/needle length was approximately 60 µm in the A1 alloy, which decreased to approximately ~20 µm in the E1 alloy. Similar results were also found in recent studies [22,23], where Ca addition reduced the length of iron-bearing phases from 30.2 to 3.8 in A356 and AlSi10MnMg alloys. These iron-bearing phases form on the oxide inclusions that are the most abundant solid particles in aluminum alloy melt [19]. Moreover, higher Fe in the Al–Si alloys decreased the size of Si eutectic phases [20]. This refinement was related to the fact that higher iron-bearing phases consume most of the potent nucleating oxide particles (as it is believed that AlP nucleates at these oxides before acting as nucleation sites for eutectic Si phases) and therefore forced Si eutectic to solidify at lower temperature and hence caused refinement [19,20]. A similar mechanism is expected to have happened in the current study where Ca-containing intermetallic compounds consumed most of the oxides and therefore caused the iron-bearing phases to solidify at lower temperature leading to a significant decrease in the size of these phases. 

**Figure 8:**
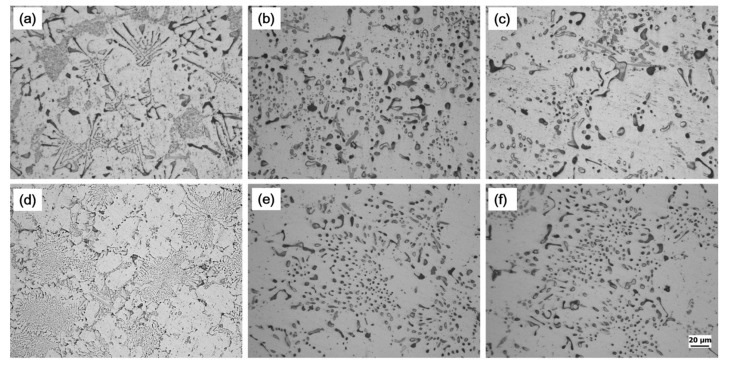
Optical microstructures of examined alloys in different temper conditions; (**a**–**c**) are respective microstructure of A1 alloy in as-cast-, SHT-, and T6-temper, whereas (**d**–**f**) are showing the microstructure of E1 alloy in as-cast-, SHT-, and T6-temper, respectively.

**Figure 9:**
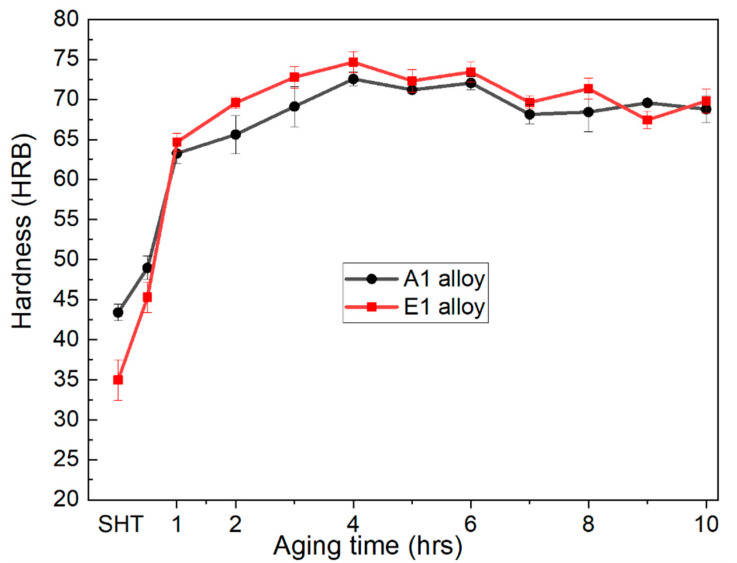
Change in the hardness values with respect to aging time for both examined alloys.

Figure 8 shows the microstructures of the examined alloys in the as-cast, as-quenched, and in T6-tempers. The interconnected structure of eutectic Al–Mg_2_Si binary and ternary eutectic Al–Mg_2_Si–Si ternary phases (Figure 8a,d) decomposed upon subjecting them to solution treatment in Figure 8b,e. The heat treatment process resulted in the breakage of coarse lamellar (Figure 8a) and fibrous eutectic structure (Figure 8d) and induced the formation of irregularly shaped Mg_2_Si remnant phases in these examined alloys. This shows that the microstructure modification of hypo-eutectic Al–Mg_2_Si ternary alloys through heat treatment is possible. Moreover, if the microstructure of the as-quenched Ca-added examined alloy is compared with the microstructure of the unmodified alloy in the as-quenched state, the size of the remnant phases was relatively smaller in the former alloy than that in A1 alloy. This can be related to the initial refined microstructure of the modified alloy. The change in hardness values versus aging time in the form of graphs for A1 alloy and E1 alloy is shown in Figure 9. There was a substantial difference in the hardness of these two alloys in as-quenched states, which were 43 HBR and 35 HBR for A1 alloy and E1 alloy, respectively. However, after just 1 h of aging treatment, a significant increase in both alloys’ hardness was observed (respective values of 63 and 64 for A1 alloy and E1 alloy). After that, there was a gradual increase in the hardness of both alloys up to the peak hardness, which was achieved after 4 h of aging treatment for both alloys. E1 alloy had shown the peak hardness of 75 HBR, which was slightly above the peak hardness value posted by A1 alloy, 73 HBR. Following this, the hardness values of both alloys decreased slightly and started fluctuating between 68 and 72 through the rest of the aging process. The aging treatment process revealed that Ca addition did not influence the kinetics of precipitation hardening notably in E1 alloy compared to that in A1 alloy. Moreover, there were no noticeable changes in the size of remnant phases upon aging treatment for 4 h (Figure 8c,f), and optical micrographs of the T6 temper were almost similar to as-quenched ones for both alloys.

**Figure 10:**
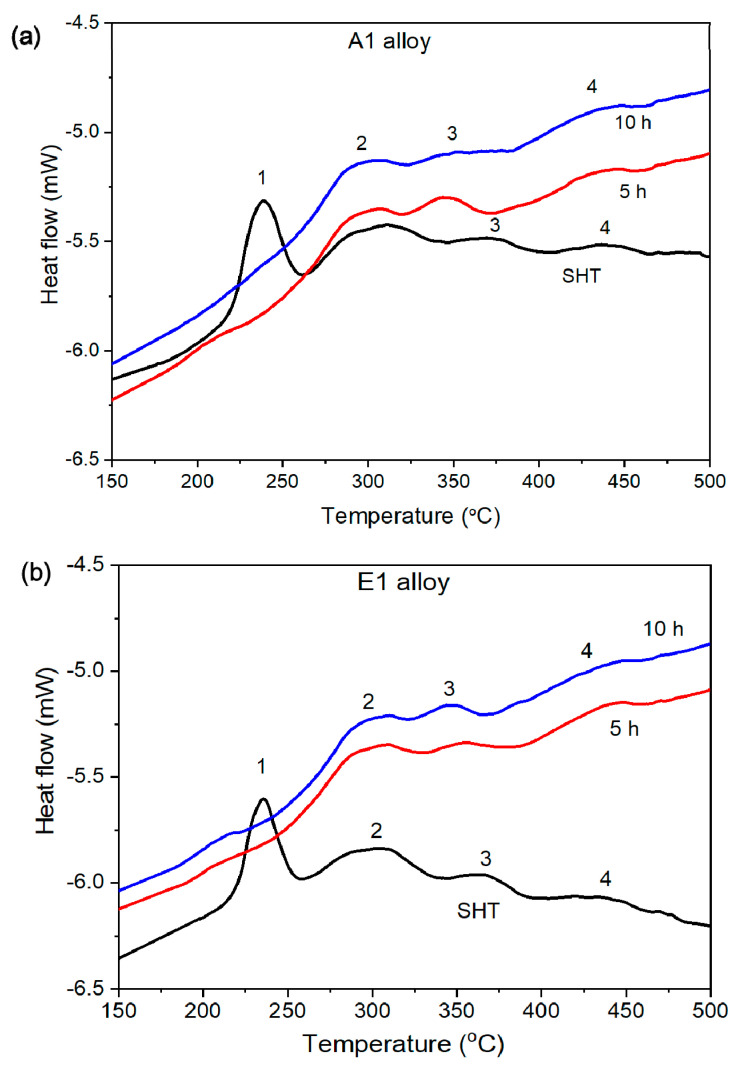
DSC curves of A1 alloy (**a**) and E1 alloy (**b**) in as-quenched (SHT), in peak hardness (5 h) and overaged (10 h) conditions.

Figure 10 shows the results of the DSC analysis of the examined alloys. A DSC analysis was a useful technique to study the precipitation hardening in the current alloys, where a significant amount of remnant Mg_2_Si and Si precipitates were present in SHT condition [28,29,30,31,32,33,34,35]. One of the benefits of DSC is its capability to detect minute changes in the microstructure during the heat treatment process. There were four dominant exothermic peaks observed in DSC curves of both the investigated alloys in as-quenched conditions (Figure 10a,b). The first peak was observed at ~240 degrees (peak 1), the second at ~300 (peak 2), the third at ~360 (peak 3), and the fourth at ~420 (peak 4). The first three peaks are related to the precipitation of Mg_2_Si phases. Similar peaks were reported in the DSC study of 6xx.x series alloys in the past studies [28,29,30,31,32,33]. Peak 1 is believed to represent the formation of the β″ metastable phase. Peak 2 and peak 3 are attributed to the formation of β′ metastable phase and β-Mg_2_Si phases that are formed because of the evolution of the β″ phase. In addition to peaks related to Mg_2_Si precipitates, one other peak was observed (peak 4), which was not reported before in Al-6xxx.x series alloys. However, one of the studies related to Al–Si binary alloy reported similar peaks attributed to precipitate Si phases [28]. Therefore, peak 4 is related to the precipitation of Si phases in the current alloys. After aging for 4 h, peak 1 disappeared completely in both alloys (Figure 10a,b), which means by the time samples reached peak hardness condition, the formation of β″ phase was almost completed. However, no endothermic peak was observed for the β″ phase, and it is because, instead of dissolution, it goes through a phase transition to more stable phases (β′ or/and β-Mg_2_Si) [34]. However, the rest of the peaks were still present even after 4 h of aging treatment, which means that the formation of these precipitates was not completed yet. With a longer aging time of 10 h, there was no difference in the DSC curves of these alloys with that of 5 h aged samples.

**Figure 11:**
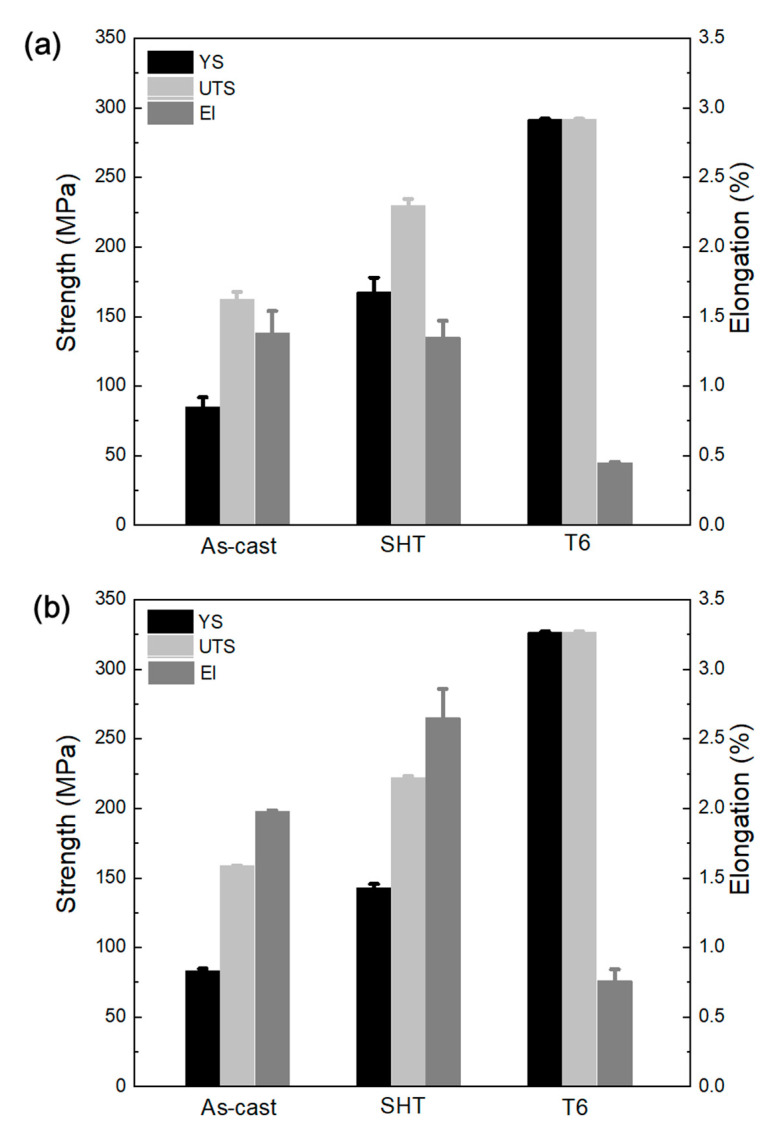
Tensile properties of (**a**) A1 alloy and (**b**) E1 alloy in different temper conditions.

The tensile properties of A1 alloy and E1 alloy in different tempers are shown in Figure 11 and also in Table 3. In the as-cast condition, the yield strength, tensile strength, and elongation of the A1 alloy were 85 MPa, 162 MPa, and 1.4%, respectively (Figure 11a). Upon the addition of 0.05%Ca, the tensile strength of the E1 alloy remained almost the same; however, it had relatively higher elongation than the base alloy (Figure 11b). When these alloys were subjected to the solution treatment process (in SHT temper), an increase in the yield strength, ultimate tensile strength, and elongation resulted. This can be attributed to the breaking of the brittle path of the binary and ternary eutectic phases and the distribution of the Mg_2_Si and Si particles in the Al-matrix (Figure 8). Moreover, the yield strength of A1 alloy was significantly higher than E1 alloy in SHT condition, but it had shown lower elongation than that of E1 alloy. The aging treatment for 4 h at 190 °C led to a significant increase in the tensile strength of A1 alloy and E1 alloys, where the tensile strength of these alloys increased to 291 and 327 MPa, respectively. However, this enhancement in tensile strength was achieved at the expense of ductility, which drastically decreased to below 1%. Nevertheless, E1 alloy has shown relatively better ductility of 0.76% than that of A1 alloy, which was ~0.45%. In fact, E1 alloy (Figure 11b) had shown higher elongation than that of A1 alloy (Figure 11a) in all temper conditions. In the Al–Si and Al–Mg–Si-based alloys, usually sharp needle-like iron-bearing phases are considered to be the cause of the fracture, and therefore, refinement or changing the morphology of this iron-bearing is reported to increase the elongation [13,19,36,37]. Similarly, the refinement of dendritic structure also improves the tensile properties and ductility of metal alloys. However, in aluminum alloys, it is reported that the decrease in the size of SDAS usually only improves elongation, and no substantial increase in the yield strength is observed [19,20]. Therefore, the improvement in elongation in E1 alloy can be attributed to the combined effect of smaller dendritic arms and refining of binary eutectic and iron-bearing phases. Moreover, enhanced elongation due to this microstructure refinement can be the reason for the higher tensile strength of E1 alloy than that of A1 alloy in T6-temper.

Table 4 shows the tensile properties of currently studied alloys along with A390 (from reference [36]) and SiC-based aluminum composites (from reference [2]). The currently studied alloys have achieved a similar level of tensile strength as those achieved in commercial A390 alloy and SiC-based composites. A390 alloy is widely used to manufacture air conditioning compressors, air compressor bodies, master brake cylinders, pumps, and other components in automatic transmission. However, poor machinability and higher shrinkage tendency due to the longer freezing range are considered problems to deal with while processing A390 alloy [26]. On the other hand, as mentioned above, processing of SiC-based composites via the casting route is difficult, and it cannot be used for complicated shapes and thinner sections because of lower fluidity [3]. Therefore, the currently developed alloys can replace these commercial materials. Moreover, the tensile strength and elongation of these newly developed alloys can be further enhanced through compositional variations with respect to Mg and Si content and other elements.

## 4. Conclusions

The microstructure of the unmodified alloy consisted of primary Al-, primary Mg_2_Si-, binary eutectic Al–Mg_2_Si-, ternary eutectic Al–Mg_2_Si–Si-, and iron-bearing- phases. This microstructure was refined significantly upon the addition of 0.05 wt% Ca.

In addition to refinement, In addition to refinement, lamellar to fibrous-type modification of binary eutectic Al–Mg_2_Si phases was also achieved in Ca-added (modified) alloy. This modification was related to increasing Ca-based intermetallics/compounds in the modified alloy that acted as nucleation sites for binary eutectic Al–Mg_2_Si phases.

The dendritic refinement with Ca addition is related to the fact that it improves the efficacy of Ti-based particles (TiAl_3_ and TiB_2_) in the melt to act as nucleation sites. The formation of Ca-based phases on oxide bifilms is believed to force the iron-bearing phases (as iron-bearing phase nucleates at oxide films) to solidify at lower temperatures and reduce their size.

Almost similar peak hardness was achieved for both alloys after 4 h of aging treatment at 190 °C. DSC analysis confirmed the formation of β″-metastable phase and its transition to β′ and finally to β-Mg_2_Si during aging treatment. Additionally, the precipitation of the Si phase was also observed.

At peak hardness, a tensile strength of 291 and El% of 0.76% and 327 MPa with El% of 0.45% was achieved for A1 alloy and E1 alloy, respectively. The higher tensile strength of E1 alloy than that of A1 alloy was attributed to the higher elongation of the former alloy, which in turn, was related to refined dendritic structure and second phases.

## Figures and Tables

**Table 1:** Chemical composition of examined alloys.

Alloy ID	Alloy Composition in Mass%				
	Mg	Si	Ti	Ca	Al
A1 (unmodified)	6	5	0.15	0.0014	Bal.
E1 (modified)	6	5	0.15	0.05	Bal.

**Table 2:** Chemical composition of Al–25 mass%Si master alloy.

Nominal Composition (mass%)	Alloy Composition in Mass%								
	Si	Fe	Cu	Mn	Ni	Na	Cr	Ca	Al
Al75–Si25	>25	0.11	0.02	0.11	0.04	0.01	0.02	0.03	Bal.

**Table 3:** Tensile properties of examined alloys in different tempers.

Temper	Alloy	YS (MPa)	UTS (MPa)	El%
As-cast	A1	85.0 ± 7.0	162.3 ± 5.4	1.38 ± 0.16
	E1	83.2 ± 1.6	159 ± 0.07	1.98 ± 0.00
SHT	A1	167 ± 10.6	229.5 ± 4.9	1.35 ± 0.12
	E1	143 ± 2.63	222 ± 1.4	2.65 ± 0.21
T6	A1	291.5 ± 0.7	291.5 ± 0.7	0.45 ± 0.0
	E1	326.5 ± 0.7	326.5 ± 0.7	0.76 ± 0.0

Here, YS, UTS, and El are referring to yield strength, ultimate tensile strength, and elongation, respectively.

**Table 4:** Comparison of the tensile properties of examined alloys with existing studied and commercial alloys.

Materials	Processing Route	YS (MPa)	UTS (MPa)	El%
A1 (T6)	PM casting	285	285	0.43
E1 (T6)	PM casting	335	335	0.76
390 (T6) ref. [36]	PM casting	310	310	<1.0
Al-9Si-0.5Mg + 20 vol.% SiC ref. [2]	PM casting	338	359	0.4
A356 + 10 vol.% SiC ref. [2]	PM casting	283	303	0.6
A356 + 15 vol.% SiC ref. [2]	PM casting	324	331	0.3
A356 + 20 vol.% SiC ref. [2]	PM casting	331	352	0.4

Here, YS, UTS, and El are referring to yield strength, ultimate tensile strength, and elongation, respectively.

## Data Availability

Available upon request from the corresponding author.
